# Coronary CT Angiography in the Quantitative Assessment of Coronary Plaques

**DOI:** 10.1155/2014/346380

**Published:** 2014-08-05

**Authors:** Zhonghua Sun, Lei Xu

**Affiliations:** ^1^Discipline of Medical Imaging, Department of Imaging and Applied Physics, Curtin University, Perth, WA 6845, Australia; ^2^Department of Radiology, Beijing Anzhen Hospital, Capital Medical University, No. 2, Anzhen Road, Chaoyang District, Beijing 100029, China

## Abstract

Coronary computed tomography angiography (CCTA) has been recently evaluated for its ability to assess coronary plaque characteristics, including plaque composition. Identification of the relationship between plaque composition by CCTA and patient clinical presentations may provide insight into the pathophysiology of coronary artery plaque, thus assisting identification of vulnerable plaques which are associated with the development of acute coronary syndrome. CCTA-generated 3D visualizations allow evaluation of both coronary lesions and lumen changes, which are considered to enhance the diagnostic performance of CCTA. The purpose of this review is to discuss the recent developments that have occurred in the field of CCTA with regard to its diagnostic accuracy in the quantitative assessment of coronary plaques, with a focus on the characterization of plaque components and identification of vulnerable plaques.

## 1. Introduction

Invasive coronary angiography is considered the gold standard for the diagnosis of coronary artery disease since it provides an excellent visualization of coronary lumen change and a roadmap of the entire coronary tree. However, invasive coronary angiography is an invasive procedure with associated morbidity and mortality. Furthermore, coronary angiography can only provide a two-dimensional outline of the coronary lumen and cannot thoroughly demonstrate the complex nature of atherosclerotic plaques which are responsible for association between the angiographic findings and clinical outcome [1]. It has been reported that almost two-thirds of acute coronary events occur in noncritical lesions (those with less than 50% lumen stenosis), highlighting the necessity for complementary imaging modalities to characterize plaque composition and to detect vulnerable plaques [2].

The noninvasive characterization of plaque morphology has potential clinical significance, since autopsy studies in coronary death have indicated that the site of coronary thrombosis is often associated with plaque rupture at sites with a thin fibrous plaque cap and a large lipid core [3–5]. Intravascular ultrasound (IVUS) is a widely used invasive imaging modality with high diagnostic accuracy for detection and quantification of coronary artery disease (CAD). IVUS allows assessment of vessel cross-sectional areas which are closely correlated with hemodynamically significant coronary stenoses [6–8]. IVUS has also been used to monitor the progression or regression of coronary plaque volume in response to clinical therapies [9, 10]. Although IVUS is the standard reference for the assessment of coronary plaque composition and progression in clinical studies [11, 12], it is an invasive procedure which is not commonly performed in routine clinical practice and thus may be limited to research studies. Coronary CT angiography (CCTA) has been widely used as an effective less invasive imaging modality to diagnose CAD [13–17]. Further, CCTA allows evaluation of coronary plaque characteristics, including plaque compositions [18]. Classification of coronary plaque compositions by CCTA has important clinical implications, with significant association of plaque components with myocardial ischemia and prediction of adverse cardiac events and prognosis [19–22]. The purpose of this review is to provide an overview of the diagnostic applications of CCTA in the quantitative assessment of coronary plaques. We focus our discussion on issues related to diagnostic accuracy, identification of vulnerable plaques, and 3D characterization of coronary plaques.

## 2. Definition of Vulnerable or High-Risk Plaques

The vulnerable atherosclerotic plaque is called a “high-risk” or “thrombosis-prone” plaque. Major criteria to characterize such plaques include the presence of active inflammation, a thin inflamed fibrous cap (<65 *μ*m) covering a lipid-rich necrotic core (>40% of the total volume of the plaque), the presence of endothelial denudation with superficial platelet aggregation, and the presence of hemodynamically significant stenosis (>90%) [23]. A more clinical relevant definition of a vulnerable plaque is a lesion that places a patient at risk for developing future major adverse cardiac events, including death, myocardial infarction, or progressive angina. The identification of such plaques before they become symptomatic would enable prognostic stratification and facilitate primary prevention (e.g., aspirin, statins, and risk factor modification).

The concept of vulnerable plaque or high-risk plaque originated from data of several studies that analyzed angiograms postthrombolytic therapy or through analysis of serial angiographic data before and after ST-elevation acute myocardial infarction [2, 27, 28]. These studies showed that most infarctions were caused by lesions that were less than severely narrowed in coronary lumen. Yamagishi et al. reported that after thrombolytic therapy the actual size of the plaque responsible for the infarction was only moderate, and the remaining luminal narrowing was related to residual thrombus formation [29]. Due to positive remodelling, the lumen remains relatively uncompromised until the plaque has grown to a larger volume [27]. Thus, in most cases, these plaques are clinically silent before the acute cardiac events occur. This emphasizes the importance of detecting vulnerable atherosclerotic plaques.

Large lipid core and calcified areas (defined as >10% of the plaque area) and thin-cap fibroatheroma have been found to be associated with positive vascular remodelling [30–32]. Clinical and biomechanical studies have explored the risk factors associated with plaque vulnerability and have identified plaque composition and morphology as key determining factors for plaque vulnerability and likelihood of rupture [33–35]. Longitudinal clinical studies indicate a strong correlation between quantitative analysis of plaque volume and composition and prediction of future cardiac events. According to a retrospective study of 1,059 patients with stable chest pain, the coronary plaque volume was found to be significantly larger in patients who developed acute coronary syndromes (ACS) than those who did not have ACS over a follow-up of 27 ± 10 months (134.9 ± 14.1 mm^3^ versus 57.8 ± 5.7 mm^3^) [36]. Another study published in 2013 investigated the additional value of using CCTA-derived semiautomatic plaque quantification in 1,650 patients with a mean follow-up of 26 ± 10 months for ACS [37]. Similarly, the ACS patients had higher total plaque volume (median 94 mm^3^ versus 29 mm^3^) and total noncalcified volume (28 mm^3^ versus 4 mm^3^) than those who did not develop ACS. Specifically, the volume of nonobstructive noncalcified plaques has been reported to be a strong indicator of predicting future cardiac events [38].

In the following sections, a critical appraisal of the literature is given to the diagnostic value of CCTA in the quantitative analysis of coronary plaques with regard to the diagnostic value, assessment of plaque vulnerability, and 3D characterization of plaques.

## 3. Coronary CT Angiography Quantitative Assessment of Plaques

It has become well established that plaque composition is a key determinant of plaque stability [35, 39]. High-risk plaques tend to be large, demonstrate positive remodelling, and have a large lipid core that occupies 40% or more of the plaque volume. A large plaque size and a larger extent of positive remodelling are prone to rupture and develop intraplaque hemorrhage in comparison to stable plaques. Thus, the ability to noninvasively detect and analyse these plaques at early stages, especially in asymptomatic and low-risk patients, would improve risk stratification without the need for more invasive procedures [35]. The development of high-speed CT allows noninvasive coronary artery visualization, detection of lumen stenosis, characterization and quantification of atherosclerotic coronary plaque, and even coronary risk stratification [33, 40].

Rapid developments in the CT technique, in particular, the increasingly worldwide use of CCTA, have demonstrated that CCTA has the potential to revolutionize how patients are risk-stratified by identifying rupture-prone, noncalcified, or mixed coronary plaques accurately and reliably. As our understanding of plaque histology has improved, there has been an increasing interest in the ability of CCTA to identify particularly the high-risk or vulnerable plaques [41, 42]. According to the SCCT (Society of Cardiovascular Computed Tomography) criteria of subdividing plaques into noncalcified, calcified, and partially calcified types, coronary CT angiography shows excellent inter- and intraobserver agreement [43]. Early comparisons of CCTA with intravascular ultrasound showed promise for characterization and quantification of plaques, although results were affected by limited scan resolution and reproducibility [44–46]. Improvements in CT technology in recent studies using 64- and post-64-slice CT have led to improved ability of CCTA to quantify and distinguish plaque types.

### 3.1. Literature Review Based on Single-Center Experiences

Pflederer et al. analysed morphological features of coronary plaques in 55 patients with acute coronary syndrome using dual-source CT angiography in comparison to 55 patients as the control group with stable angina pectoris [47]. Their results showed that culprit lesions in acute coronary syndrome show specific morphologic characteristics which are characterized by larger plaque volumes, higher remodelling indices, greater proportions of noncalcified material, and presence of spotty calcification. Motoyama et al. in their large retrospective study showed that plaques with positive vascular remodelling and containing large areas of low attenuation assessed by CCTA are associated with a higher risk of acute coronary syndrome when compared with patients without these characteristics (22.2% versus 0.5%) [36]. These CT characteristics confirm features of unstable plaques which were previously reported by pathology.

A head to head comparison of CCTA with IVUS further confirms the diagnostic accuracy of CCTA in the quantitative assessment of coronary plaques. Nakazato et al. performed a direct comparison between CCTA and IVUS with regard to the quantification and characterization of coronary plaque volume and adverse plaque features in 27 consecutive patients with a total of 30 individual coronary plaques [48]. They observed a high correlation between total plaque volumes as quantified by CCTA in comparison to IVUS with no significant differences between the two methods. Bland-Altman limits of agreement for vessel volume, lumen volume, and plaque volume ranged from −53.7 to 53.1 mm^3^ with a bias of −0.3 mm^3^, −24.1 to 32.2 mm^3^ with a bias of 4.0 mm^3^, and −40.6 to 31.8 mm^3^ with a bias of −4.4 mm^3^, respectively. Furthermore, their results showed that CCTA has high diagnostic accuracy for identification of adverse plaque characteristics (low-attenuation plaque, positive remodelling, and spotty calcification) with no significant differences between CCTA and IVUS. Similarly, Papadopoulou et al. compared 64-slice CT angiography with IVUS in 32 patients consisting of 44 coronary arteries which were coregistered for quantitative analysis of the diagnostic performance of CCTA [49]. They analysed 1364 coregistered 1 mm cross-sections and 255 segments of 5 mm length. The sensitivity and specificity of CCTA were 86% and 71% at cross-sectional level assessment and 96% and 88% at segment level assessment, respectively. A significantly strong correlation was found between CCTA and IVUS in the quantification of plaque volumes. CCTA demonstrated underestimation of the plaque volume of noncalcified and overestimation of mixed/calcified plaque, although this was not significantly different from those measured by IVUS.

The ATLANTA (assessment of tissue characteristics, lesion morphology, and hemodynamics by angiography with fractional flow reserve, intravascular ultrasound and virtual histology, and noninvasive computed tomography in atherosclerotic plaques) study was a prospective, single-center study with the aim of determining if CCTA-derived measurements of plaque components correlated well with IVUS/VH (virtual histology) measurements [50]. Fifty lesions in 50 patients were analysed in this study with all of the patients undergoing invasive angiography, CCTA, and IVUS/VH examinations. A significant correlation was found between CCTA and IVUS/VH in terms of plaque geometric, compositional, lumen area and vessel area measurements. This study adds to the current literature on the accuracy of CCTA for coronary plaque quantification as it is the first study that used a multimodality approach involving 3 morphological methods to assess coronary lesions.

### 3.2. Literature Review Based on Multicenter Trials

The ACCURACY (assessment by coronary computed tomographic angiography of individuals undergoing invasive coronary angiography) study is currently the only available multicenter trial involving 16 centers which was designed to determine the relationship between coronary plaque composition as detected by CCTA and lumen diameter stenosis as quantified by invasive coronary angiography [51]. This prospective study involved 230 patients with obstructive CAD diagnosed in 38.7% of patients (>50% lumen stenosis). Results of the ACCURACY trial demonstrate a strong association between the presence of mixed plaque composition and obstructive CAD at a per-segment and per-patient level assessment. In contrast, coronary artery segments with calcified plaques are rarely associated with obstructive coronary stenosis. This is consistent with those reported by single centers [36, 52], highlighting the prognostic significance of plaque composition characterization, although more studies based on multicenter trials are necessary to confirm these findings.

### 3.3. Literature Review Based on Systematic Review and Meta-Analysis

There are one systematic review and three meta-analysis reports available in the literature with regard to the diagnostic value of CCTA in detecting coronary plaques [53–56]. Springer and Dewey in their early systematic review reported that CCTA has the potential to become a useful diagnostic tool for the analysis of coronary plaques when compared to IVUS, based an analysis of 9 studies [53]. Their results were limited by the small sample sizes and methodological flaws in the studies reviewed. Recent reports with inclusion of more studies have confirmed the reliable diagnostic accuracy of CCTA for detection of coronary plaques [54–56].

Gao et al. conducted a meta-analysis of 17 articles by comparing CCTA with IVUS with regard to the diagnostic accuracy of CT in detecting coronary plaques [54]. They observed a good weighted sensitivity of 92% and specificity of 93% for CCTA in the detection of any type of coronary plaque as compared with IVUS and a good positive and negative predictive value (>84%) for diagnosis of any plaque in a wide range of pretest probabilities. They recommended that CT be considered the prime noninvasive alterative to intravascular ultrasound for detecting coronary plaques.

Voros et al. conducted a meta-analysis of CCTA against IVUS for the assessment of coronary vessel, plaque sizes, and the diagnostic accuracy [55]. The authors identified 20 studies eligible for assessment of diagnostic accuracy of CCTA to detect plaques and 22 studies for comparison of vessel and plaque dimensions. CCTA has been shown to demonstrate excellent diagnostic accuracy for the detection of coronary plaques when compared to the reference standard, IVUS, with the pooled sensitivity and specificity of 90% (95% confidence interval [CI]: 83% to 94%) and 92% (95% CI: 90% to 93%). Plaque area, volume, and area stenosis measurements were similar between CCTA and IVUS, while CCTA slightly overestimated luminal area. This is also confirmed by a recently published meta-analysis comparing CCTA with IVUS for quantitative assessment of coronary stenosis and plaque burden [56]. Forty-two studies consisting of 1,360 patients were identified in this analysis with results showing no significant difference between CCTA and IVUS measurements of vessel cross-sectional area, plaque area, percentage of area stenosis, or plaque volume. The sensitivity and specificity of CCTA to detect any plaque were 93% and 92% when compared to IVUS, indicating that CCTA is accurate for the quantification of coronary plaques.

In summary, CCTA not only allows accurate detection of coronary atherosclerotic plaques but also enables quantitative analysis of plaque vessel area, lumen area, and plaque burden. Attempts at plaque quantification and characterisation using coronary CT angiography have been successful, although further refinements regarding reproducibility, accuracy, and ability to predict future cardiac events are required. With further improvements in CT imaging processing and diagnostic value, coronary CT angiography will become an integral part of the routine diagnostic approach for the noninvasive detection of vulnerable plaques that are prone to rupture so that appropriate preventive strategies can be implemented in a targeted population.

## 4. Detection of Coronary Plaque Composition to Determine Plaque Vulnerability

Specific plaque features demonstrated by CCTA have been linked with ACS and other adverse cardiac events. These “adverse” plaque features include the low-attenuation plaque, remodelling index, spotty calcification, and the “napkin-ring” sign. The low-attenuation plaque refers to focal noncalcified plaque, within a specific low-attenuation threshold range between 30 and 60 HU [36, 52]. The remodelling index is defined as the ratio of the maximum vessel area (or diameter) to a normal reference vessel area (or diameter) as a measure of coronary arterial remodelling, and plaques are classified as having positive remodelling when the remodelling index is >1.0 [57–59]. Spotty calcifications are defined by IVUS as small calcified nodules with length <3 mm within an IVUS arc of ≤90° [60, 61]. The napkin-ring sign, or the presence of a “contrast rim,” has recently been reported to be a CT signature of high-risk coronary atherosclerotic plaque [62, 63].

### 4.1. Low-Attenuation Area

A major focus of current research is targeted toward identification of vulnerable plaques destined to become index lesions [64]. This has been driven by classic pathological and imaging (mainly coronary angiographic) observations suggesting that ACS is most commonly associated with rupture of mild angiographic stenoses. The pathological features of vulnerable coronary plaques are well established, including the presence of a large necrotic core, a large volume, and positive remodelling of the vessel [65, 66].

**Figure 1:**
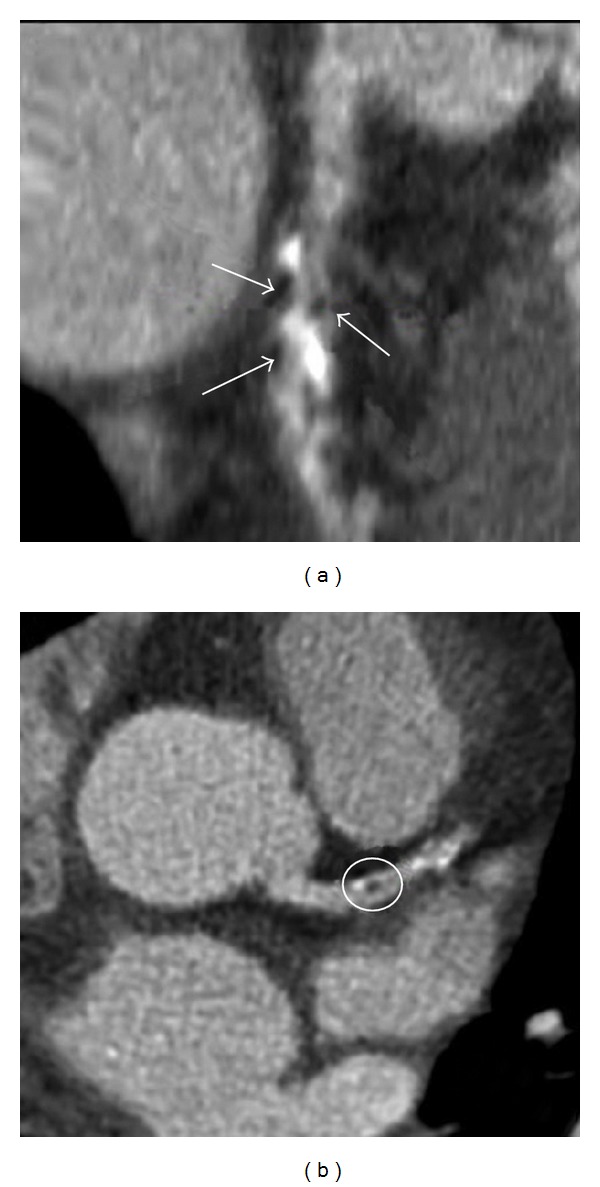
64-slice coronary CT angiography (CCTA) in a 57-year-old man with non-ST elevation myocardial infarction. (a) CCTA image of multiplanar reconstruction of left anterior descending artery (LAD) showing multiple black low-density areas (white arrows) within the plaque. (b) Visualization of the LAD stenosis showing positive remodelling and presence of low-density plaque (<30 Hounsfield units) in the middle of the plaque (black area, white circle). Reprint with permission from [24].

Studies comparing CCTA images with IVUS have shown that CCTA can accurately evaluate image characteristics in vulnerable plaques in patients with ACS. Takaoka et al. in their study compared CCTA with IVUS for analysing unstable plaques and they found that CT densities correlated with soft plaques as diagnosed by IVUS were 32.9 ± 8.7 HU and those associated with thrombus were 43.2 ± 10.7 HU, while both values were significantly lower than those of fibrous plaques as identified by IVUS, which were 82.5 ± 22.6 HU [67]. Their findings showed that CT values for plaque density are significantly lower for the soft components than for fibrous components of the atherosclerotic plaques. Benedek et al. also reported that CT characterizes the unstable coronary culprit lesion with a large total plaque volume, higher remodelling index, and larger amount of low-density atheroma or necrotic core within the plaque when compared with nonculprit lesions (Figure 1) [24]. Vulnerable plaques were found to present a significantly higher burden with the low-density plaque with 30 HU (23.3 mm^3^ versus 7.6 mm^3^) and 60 HU (33.4 mm^3^ versus 16.9 mm^3^) thresholds than nonvulnerable plaques; however, no significant difference was reached for higher density plaques (60–100 HU threshold).

The PROSPECT (providing regional observations to study predictors of events in the coronary tree) trial used multimodality intravascular imaging to identify the lesion characteristics that represent risk markers for future adverse cardiac events and concluded that lesion characteristics predictive of future events were a large plaque burden, a small luminal area, and thin-cap fibroatheroma, further confirming the importance of higher volume lesions as substrates for ACS [68].

### 4.2. Positive Remodelling

The response of coronary arteries to atherosclerosis and plaque growth manifested as either compensatory enlargement or shrinkage is referred to as coronary artery remodelling. Increase in vessel size due to outward expansion of the vessel wall is called positive remodelling, while decrease in vessel size due to vessel shrinkage is defined as negative remodelling [69]. The assessment of compensatory enlargement or positive remodelling of coronary arteries has attracted a lot of attention due to the association of positively remodelled coronary plaques with plaque vulnerability and the propensity to cause future cardiac events based on histopathological and clinical studies [70–74]. IVUS is the preferred method for the assessment of coronary artery remodelling in vivo [70–75], whereas CCTA as a less invasive imaging modality can not only visualize the coronary artery lumen but also coronary atherosclerotic plaque, with promising results reported compared with IVUS [75, 76].

**Figure 2:**
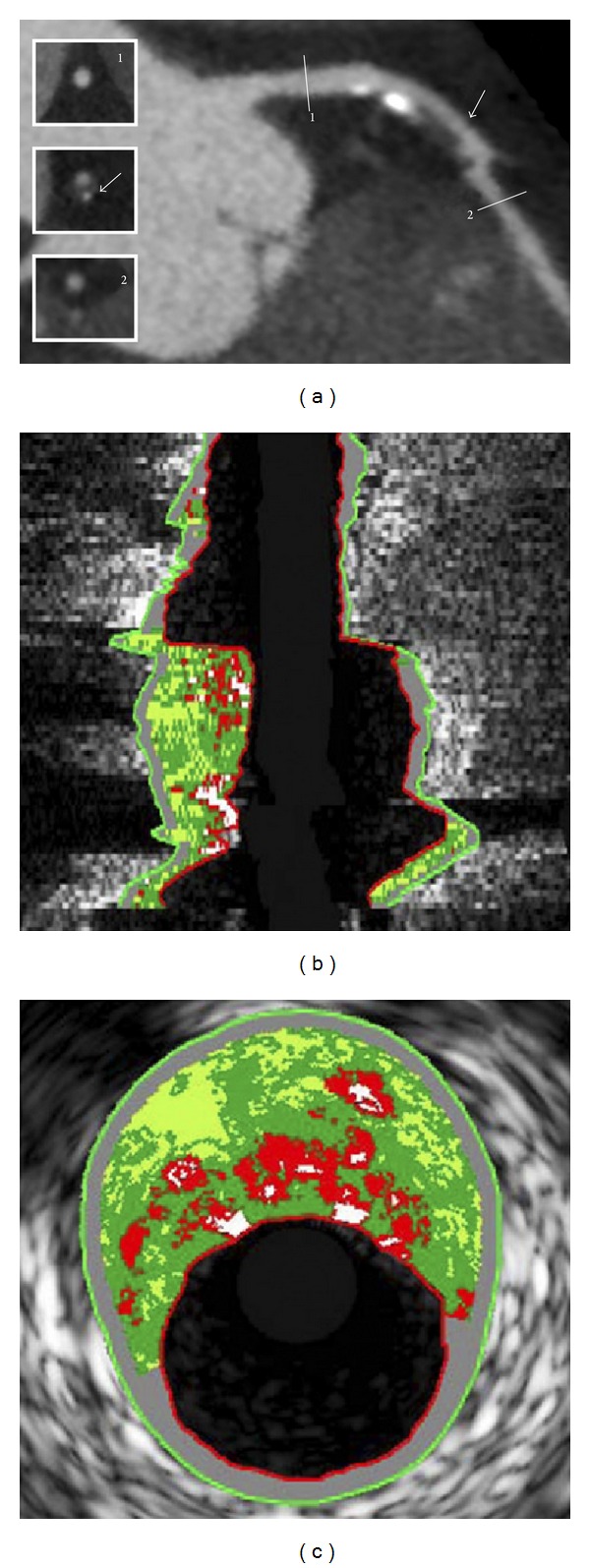
Example of lesion with positive remodelling on 320-row CT angiography and corresponding findings on virtual histologic intravascular ultrasound image. Curved multiplanar reconstruction of left anterior descending coronary artery showing a large plaque in the proximal segment of the vessel (a). The diameter of the vessel at the plaque site (arrow) is larger compared to the diameter at the reference sections (1, 2), indicating the presence of positive remodeling. Corresponding longitudinal (b) and cross-sectional (c) virtual histologic intravascular ultrasound images demonstrate an outward-remodeled lesion, with a large plaque burden (69%) and a large amount of necrotic core (19%) (red areas), corresponding to a thin-capped fibroatheroma lesion. Reprint with permission from [25].

Achenbach et al. in their early study based on 16-slice CT indicated that CCTA has potential to assess remodelling of coronary plaques in selected patients when compared to IVUS [77]. They found a lesser degree of remodelling in lesions with a significant (>50%) lumen stenosis as compared to nonstenotic coronary lesions. Latest reports confirm the diagnostic value of CCTA in the quantification of positive remodelling of coronary lesions [25, 75, 78]. CCTA with use of high resolution 64- or more slice scanners significantly improves image quality, resulting in accurate assessment of coronary lesions. Kr$\ddot{\text{o}}$ner et al. evaluated the association between positive remodelling on 64-/320-slice CCTA and plaque characteristics on IVUS/VH in 55 patients with a total of 99 plaques being identified and analysed [25]. Positive remodelling on CCTA was related to vulnerable plaque characteristics on IVUS/VH as lesions with positive remodelling on CCTA were found to have a significantly higher plaque burden (large necrotic core and thin-cap fibroatheroma) on IVUS/VH images (Figure 2). Guass et al. in their recent study explored the accuracy of dual-source CCTA for detecting coronary remodelling of atherosclerotic plaques in comparison with IVUS and their results are in line with other studies [78]. In particular, authors in this study found that a cut-off remodelling index ≥1.1 improved specificity to 78% compared with the specificity of 45% using the standard cut-off remodelling index ≥1.05, although the sensitivity reduced from 100% to 83%. They suggested that a threshold of 1.1 for the remodelling index should be used to enable accurate classification of positively remodelling plaques by CCTA.

### 4.3. Napkin-Ring Sign

Recently, several studies have described specific attenuation pattern of atherosclerotic plaques on coronary CT images characterized by a plaque core with low CT attenuation surrounded by a rim-like area of higher CT attenuation as napkin-ring sign [79–81]. A similar ring-like enhancement was found in plaques with thin-cap fibroatheromas or in patients presenting with ACS but less commonly seen in plaques without thin fibrous caps or never seen in patients with stable angina [26, 82]. Nishio et al. reported that a ring-like enhancement pattern was observed in 445 of disrupted plaques detected by angioscopy in patients with suspected ischemic heart disease but only in 6% of plaques without signs of rupture [82]. This sign has been reported to be associated with high-risk plaques and development of acute coronary syndromes [26, 82–85].

**Figure 3:**
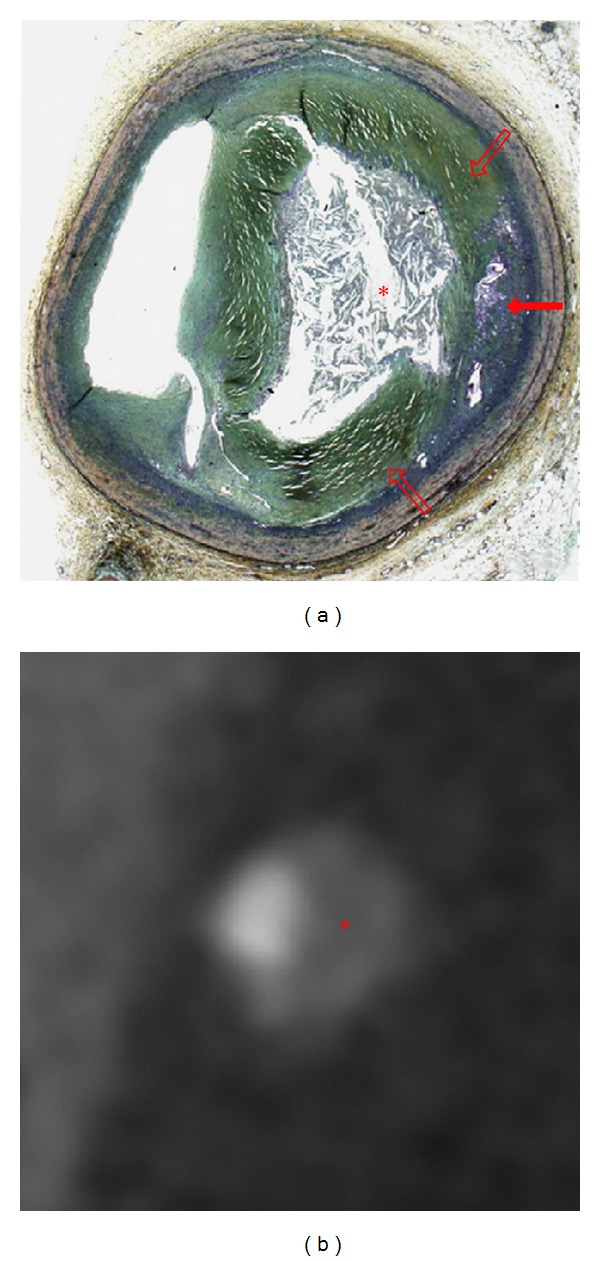
Late fibroatheroma as classified by histology (a). Note the large necrotic core (∗) in the center of the plaque, which correlates with the hypodense center of the plaque (∗) in CT (b). The core is surrounded by prominent fibrotic tissue (open arrows), which appears as a hyperdense ring around the core in CT. Thus the plaque has a ring-like appearance in coronary CT angiography which was coined as napkin-ring sign. Additionally neovascularization is present within the plaque (closed arrow). Reprint with permission from [26].

Seifarth et al. in their ex vivo study assessed histopathological features of advanced atherosclerotic plaques in 7 human donor hearts in relation to the napkin-ring sign observed in CCTA [26]. In this study, the results showed that plaques with a napkin-ring sign were associated with greater necrotic core area than plaques without this sign (1.1 mm^2^ versus 0.46 mm^2^) (Figure 3). Further, the plaque size surrounding the necrotic core was twice as large in plaques with a napkin-ring sign compared to those plaques without the sign (10.15 mm^2^ versus 6.36 mm^2^). These findings indicate that the napkin-ring sign could serve as a marker for advanced plaques on CCTA. Otsuka et al. in their prospective study consisting of 895 patients with a mean follow-up of 2.3 years further determined the predictive value of the napkin-ring sign for future ACS [84]. Plaques with a napkin-ring sign on CCTA were found to be of significant prognostic importance for ACS events, independent of other CCTA features including positive remodelling and low-attenuation plaque. Kaplan-Meier analysis showed the plaques with napkin-ring signs were associated with a higher risk of ACS events compared with those without such a sign.

In summary, the current analysis confirms that the majority of acute coronary syndromes are caused by coronary plaques with features of positive remodelling, low-attenuation area, and napkin-ring sign. The substudy of the PROSPECT trial demonstrated that vulnerable plaques are frequently seen in the proximal coronary tree, followed by the mid coronary tree and the least in the distal coronary tree [85]. Of these three characteristics of vulnerable plaques, the current evidence shows that the napkin-ring sign represents the most reliable indicator of predicting ACS. The possible mechanisms of the napkin-ring sign include intraplaque vasa vasorum enhancement by the CCTA [86, 87], intraplaque hemorrhage or thrombus with peripheral enhancement by contrast media, or microcalcifications in the plaque which are strongly associated with the development of future ACS [36]. Detection of a napkin-ring sign could help identify patients at high risk of future ACS events, although large-scale multicenter studies are needed to confirm these findings.

## 5. 3D Virtual Intravascular Endoscopy Characterization of Coronary Plaques

Coronary angioscopy allows direct visualization of the intraluminal surface and is the gold standard for assessing surface characteristics of the coronary wall in patients, such as plaque colour, presence of thrombus, and integrity of the plaque [88–90]. Coronary angioscopy can be used to identify vulnerable plaques in patients with CAD. The plaque in the coronary artery can be white or yellow angioscopically, depending on the histological presence of thick or thin fibrous cap [91]. However, some limitations that exist in angioscopy include the inherently limited ability to identify only surface morphology and its blindness to subsurface pathology and internal plaque composition [88]. Visualization of the proximal coronary artery segments is limited as the left main stem cannot be occluded and very distal arteries are not readily visualized. Finally, balloon occlusion which is required to generate clear visible field can lead to the complications of coronary ischemia. Thus, coronary angioscopy is not commonly performed in clinical practice, while virtual angioscopy or virtual endoscopy with aid of high resolution medical imaging is increasingly used as a less invasive method to generate similar endoscopic images.

CT virtual intravascular endoscopy (VIE) is a 3D imaging technique which presents intraluminal views of the anatomy of human vessels in a noninvasive way. By acquiring volumetric CT data after contrast enhancement in a single breath-hold, it is possible to render 3D images of vascular structures that simulate images obtained from conventional endoscopy/angioscopy. VIE images of the normal arterial anatomy as well as pathological changes such as calcification, arterial stenosis, aneurysm, and intraluminal thrombus can be visualized in a way that may enhance diagnostic capability compared with conventional 2D or 3D visualization methods [91–102].

**Figure 4:**
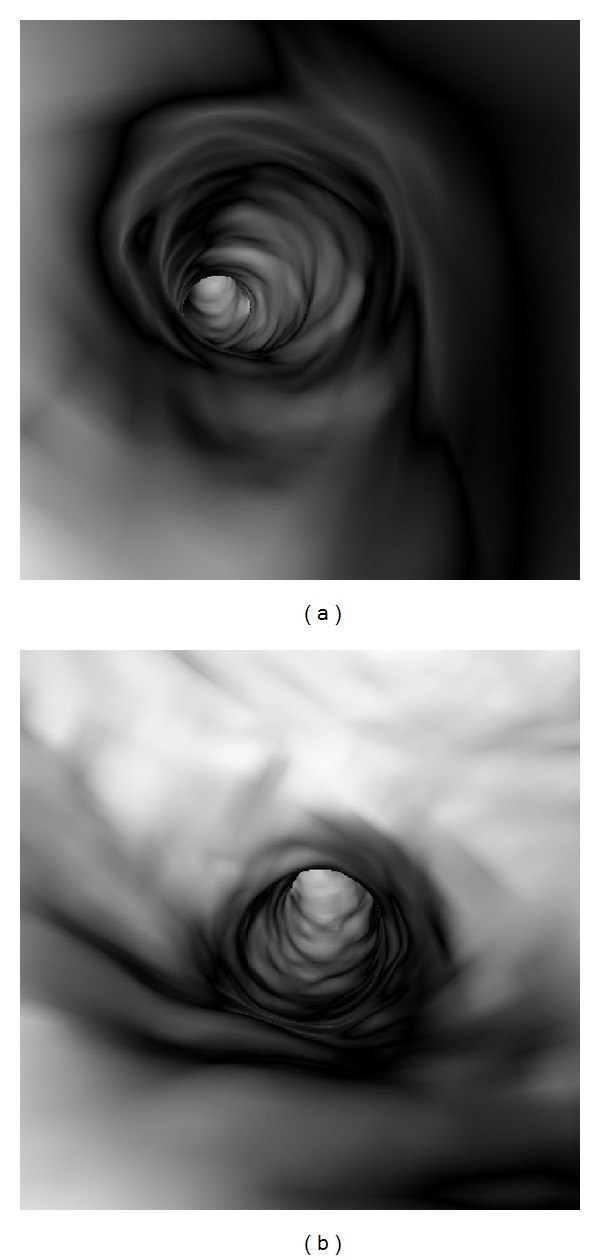
Virtual intravascular endoscopy visualization of normal coronary artery, (a) right coronary artery and (b) left coronary artery.

In normal coronary artery without presence of coronary plaques or atherosclerotic changes, the coronary wall appears smooth with clear demonstration of the ostial configuration and lumen on VIE visualization (Figure 4). When the plaques are present in the artery wall, different configurations are noticed, depending on the composition of the plaques. Studies have shown that VIE, as a novel visualization tool, clearly demonstrates the intraluminal changes of different types of plaques and offers additional information compared to conventional 2D or 3D extraluminal views [103, 104]. The appearance of coronary plaques visualized on VIE images can be classified into regular, smooth, and irregular appearance, depending on the type and composition and extent of the plaques.

### 5.1. VIE Visualization of Coronary Plaques: Smooth Intraluminal Appearance

**Figure 5:**
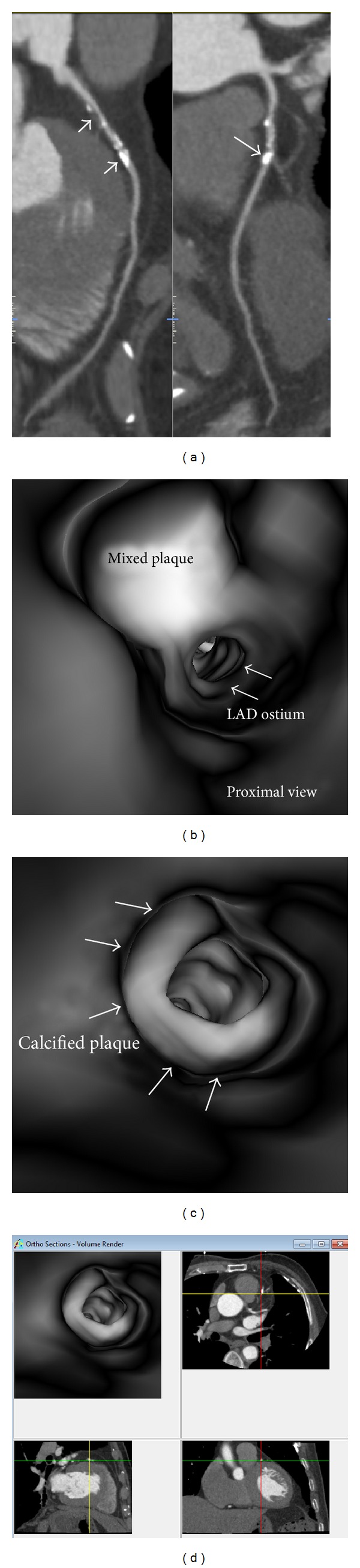
Virtual intravascular endoscopy (VIE) visualization of mixed and calcified plaques in a 75-year-old male with coronary artery disease. (a) Curved planar reformation shows mixed plaque (short arrow) and calcified plaques (long arrows) in the proximal segment of left anterior descending coronary artery (LAD). (b) Close VIE view of the mixed plaque. (c) VIE view of the calcified plaque at LAD with significant lumen stenosis. (d) Orthogonal views help to confirm the intraluminal plaque position of the calcified plaque.

**Figure 6:**
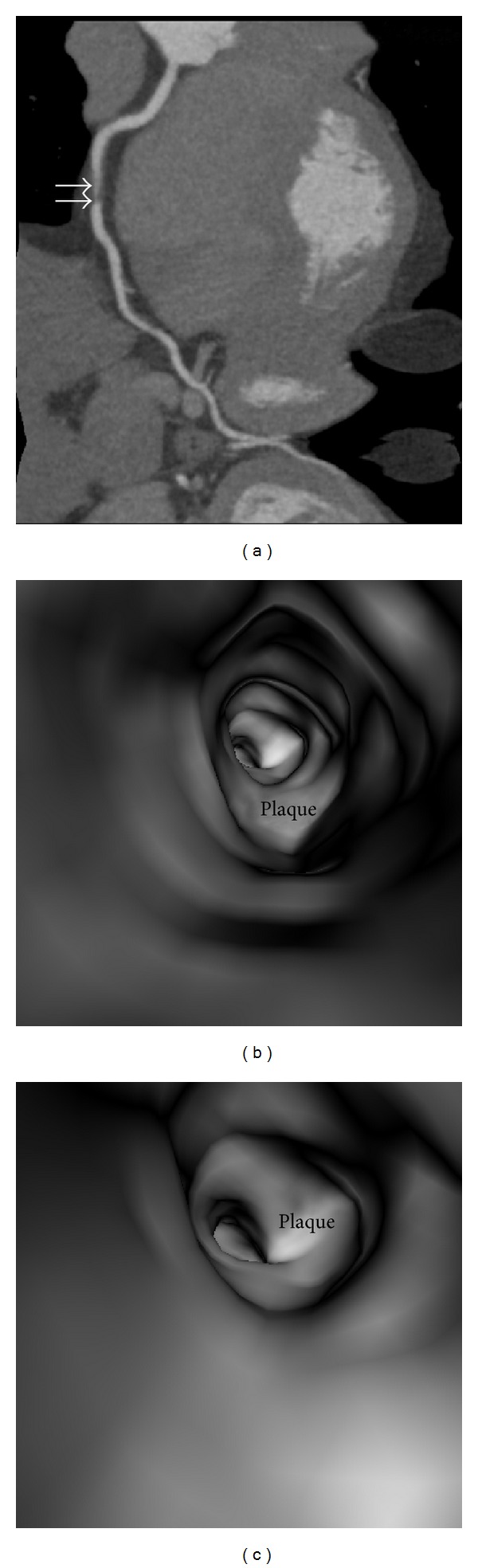
Virtual intravascular endoscopy (VIE) visualization of noncalcified plaque in a 42-year-old male with suspected coronary artery disease. (a) Curved planar reformation shows a noncalcified plaque at the proximal segment of right coronary artery (arrows). (b) and (c) VIE views of the plaque demonstrate smooth intraluminal appearance with significant stenosis.

When calcified or noncalcified plaques are present in the coronary wall, VIE shows smooth appearance in the coronary wall, with a protruding sign arising from the artery wall, resulting in the lumen stenosis, as shown in Figures 5 and 6.

### 5.2. VIE Visualization of Coronary Plaques: Irregular Intraluminal Appearance

**Figure 7:**
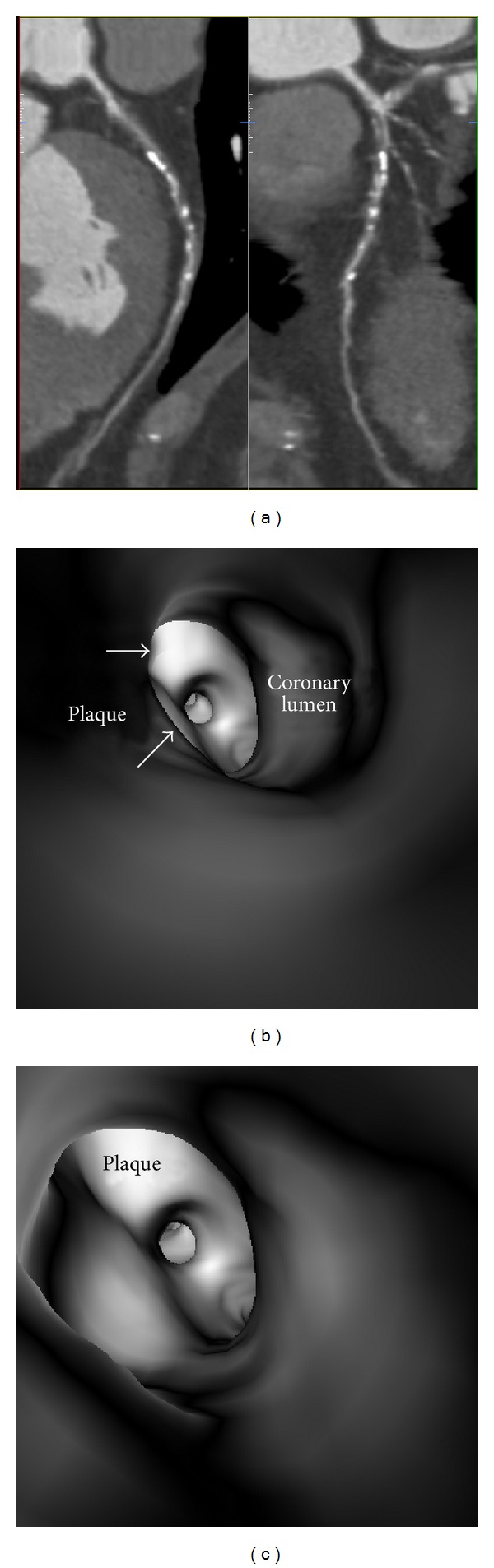
Virtual intravascular endoscopy (VIE) visualization of mixed plaque in a 56-year-old male with coronary artery disease. (a) Curved planar reformation shows mixed plaques at the proximal segment of left anterior descending artery (LAD). Corresponding VIE images (b) and (c) show that the irregular intraluminal appearance is due to presence of different plaque components within the plaque.

Irregular appearance of the coronary wall is commonly observed in the mixed plaques as shown on VIE since different components are contained within the plaques (Figure 7). This indicates that the coronary wall undergoes different stages of remodelling, consisting of both stable stage involving formation of calcified plaques and unstable period involving deposition of noncalcified plaques which contain lipid-rich components.

**Figure 8:**
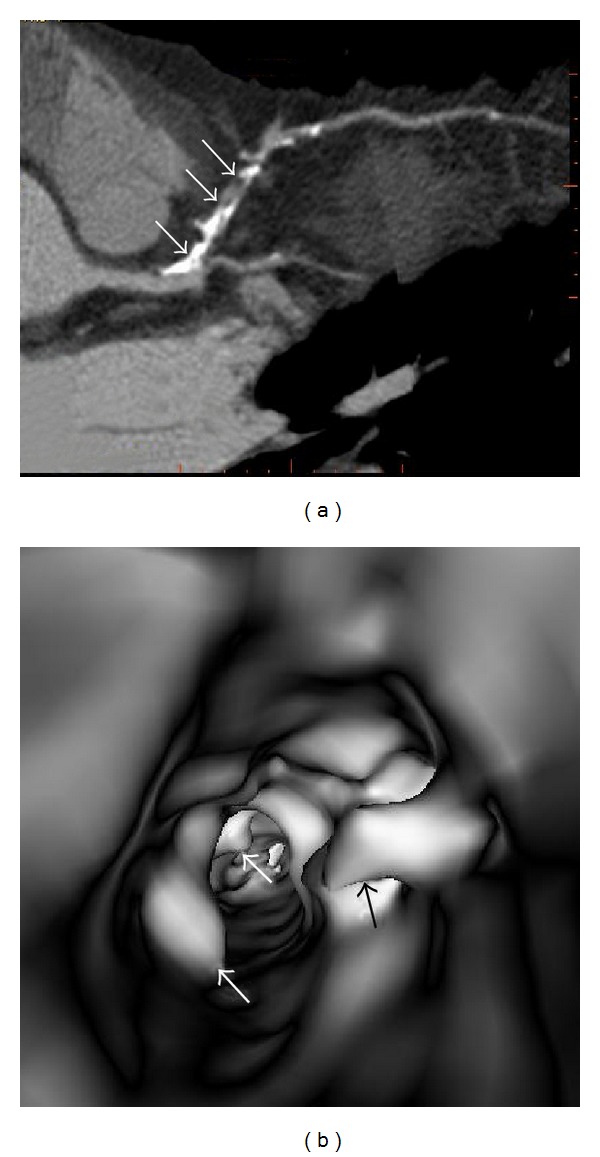
Virtual intravascular endoscopy (VIE) appearance of extensively calcified plaque in a 52-year-old man with coronary artery disease. (a) Curved planar reformation shows significant stenosis of left anterior descending coronary artery due to presence of plaques with heavy calcification (arrows). (b) VIE shows irregular intraluminal changes due to different compositions within the plaques (arrows).

Irregular appearance of the coronary wall is also seen in extensively calcified plaques due to the remodelling of the artery wall caused by plaques which are formed along the coronary artery (Figure 8), as most of the calcified plaques are eccentric. For the type of concentrically calcified plaques, smooth and regular appearance is noticed at the artery wall but with central lumen stenosis (Figure 5(c)).

In summary, 3D VIE visualization has potential applications for assessment of coronary plaques in terms of plaque characterization and intraluminal appearances. VIE findings are considered valuable for enhancing our understanding of the effect of coronary plaques on coronary wall and subsequent clinical outcomes. Further studies are needed to correlate coronary wall changes due to different types of plaques with corresponding disease progression (e.g., major adverse cardiac events) with the aim of providing risk stratification and patient management.

## 6. Summary and Concluding Remarks

Significant progress has been made in the last decade to advance our understanding of the biology of atherosclerosis. This has resulted in accurate identification of vulnerable plaques that are prone to inducing ACS, which can account for the sudden onset of clinical presentation in the majority of patients with CAD. Coronary CT angiography has been widely investigated to define the specific characteristics of vulnerable atherosclerotic plaques, with promising results having been achieved compared to intravascular ultrasound. Coronary CT angiography allows visualization of coronary atherosclerotic lesions with characteristics assumed to be associated with plaque vulnerability in patients with ACS as compared with patients with stable clinical presentation. Furthermore, coronary CT angiography enables generation of 3D intraluminal views of coronary wall and plaque with regard to different types of atherosclerotic plaques, which further strengthen CT contribution toward the differentiation of vulnerable patients. However, there exists a large variability regarding the plaque characteristics in relation to the plaque vulnerability in coronary CT angiography, making it more difficult to identify the actual culprit lesion in patients with ACS. Future research will be focusing on the ability of coronary CT angiography to investigate plaque characteristics throughout the entire coronary system rather than identifying a single lesion in a noninvasive fashion with the aim of identifying vulnerable patients at risk for ACS. Large, longitudinal, multicenter trials to determine the prognostic value of comprehensive plaque assessment and standardized CT indicators of vulnerable plaques are also warranted.
